# The Saudi Ministry of Health’s Twitter Communication Strategies and Public Engagement During the COVID-19 Pandemic: Content Analysis Study

**DOI:** 10.2196/27942

**Published:** 2021-07-12

**Authors:** Fatimah Mohammed Alhassan, Sharifah Abdullah AlDossary

**Affiliations:** 1 Department of Health Informatics College of Public Health and Health Informatics King Saud bin Abdulaziz University for Health Sciences Riyadh Saudi Arabia; 2 King Abdullah International Medical Research Center Riyadh Saudi Arabia

**Keywords:** COVID-19, Crisis and Emergency Risk Communication, effective communication, health authorities, outbreak, pandemic, public engagement, public health, social media, Twitter

## Abstract

**Background:**

During a public health crisis such as the current COVID-19 pandemic, governments and health authorities need quick and accurate methods of communicating with the public. While social media can serve as a useful tool for effective communication during disease outbreaks, few studies have elucidated how these platforms are used by the Ministry of Health (MOH) during disease outbreaks in Saudi Arabia.

**Objective:**

Guided by the Crisis and Emergency Risk Communication model, this study aimed to explore the MOH’s use of Twitter and the public’s engagement during different stages of the COVID-19 pandemic in Saudi Arabia.

**Methods:**

Tweets and corresponding likes and retweets were extracted from the official Twitter account of the MOH in Saudi Arabia for the period of January 1 through August 31, 2020. Tweets related to COVID-19 were identified; subsequently, content analysis was performed, in which tweets were coded for the following message types: risk messages, warnings, preparations, uncertainty reduction, efficacy, reassurance, and digital health responses. Public engagement was measured by examining the numbers of likes and retweets. The association between outbreak stages and types of messages was assessed, as well as the effect of these messages on public engagement.

**Results:**

The MOH posted a total of 1393 original tweets during the study period. Of the total tweets, 1293 (92.82%) were related to COVID-19, and 1217 were ultimately included in the analysis. The MOH posted the majority of its tweets (65.89%) during the initial stage of the outbreak. Accordingly, the public showed the highest level of engagement (as indicated by numbers of likes and retweets) during the initial stage. The types of messages sent by the MOH significantly differed across outbreak stages, with messages related to uncertainty reduction, reassurance, and efficacy being prevalent among all stages. Tweet content, media type, and crisis stage influenced the level of public engagement. Engagement was negatively associated with the inclusion of hyperlinks and multimedia files, while higher level of public engagement was associated with the use of hashtags. Tweets related to warnings, uncertainty reduction, and reassurance received high levels of public engagement.

**Conclusions:**

This study provides insights into the Saudi MOH’s communication strategy during the COVID-19 pandemic. Our results have implications for researchers, governments, health organizations, and practitioners with regard to their communication practices during outbreaks. To increase public engagement, governments and health authorities should consider the public’s need for information. This, in turn, could raise public awareness regarding disease outbreaks.

## Introduction

### Background

Coronaviruses are a large family of viruses that cause diseases ranging from those with common cold symptoms to more severe pneumonia-like illnesses [1]. On December 31, 2019, the World Health Organization (WHO) Country Office in China declared that a new coronavirus, SARS-CoV-2, had been detected in Wuhan. Within a few weeks, the virus had spread from Wuhan to many provinces within China. It subsequently spread outside China, reaching over 200 countries. The rapid and continuous spread of the virus led the WHO to declare COVID-19, caused by SARS-CoV-2, a public health emergency of international concern on January 30, 2020, and a pandemic on March 11, 2020 [2-5].

Saudi Arabia is the second largest Arab country with a population of over 34 million people [6]. The Ministry of Health (MOH) in Saudi Arabia is the largest provider of health care services, providing approximately 60% of the health care services nationwide, while the remainder is covered by other governmental and private facilities [7]. Since the confirmation of the first case of COVID-19 in Saudi Arabia on March 2, 2020, the government has taken prompt and decisive measures to combat the outbreak. These measures included, but were not limited to, closures of borders, schools, mosques, and Umrah (the minor pilgrimage to Mecca, which can be undertaken any time of the year); cessation of international flights; mandatory quarantine periods for returning travelers; workplace closures, with individuals working from home (apart from essential workers); and partial to complete lockdowns [8]. Digital health measures were also implemented and effectively utilized during the pandemic [9,10]. As of March 25, 2020, the MOH designated 25 hospitals with 80,000 hospital beds and 8000 intensive care unit beds for the treatment of COVID-19 cases. An additional 2200 beds were allocated for the isolation of suspected and quarantined cases [11]. Saudi Arabia has robust preparedness and response capabilities that have been strengthened through prior experience with the Middle East respiratory syndrome coronavirus and decades of planning and managing religious mass gatherings of Hajj (the annual pilgrimage to Mecca) and Umrah, which can serve as a model for other countries in the region.

In public health emergencies such as the COVID-19 pandemic, effective communication is crucial for informing the public about disease situation updates, motivating them to adopt preventive measures, and reassuring them that the government is in control of the outbreak [12-14]. Such communication requires timely dissemination of accurate and reliable information. Traditionally, governments and public health authorities have relied on websites, print media, and television as the main platforms for disseminating outbreak-related information to the public. However, the evolution of digital communications technologies such as social media has facilitated increased sharing of information for both public health authorities and the general public.

In recent years, social media has developed rapidly. Both individuals and health care organizations are using these platforms increasingly to communicate and share information [15]. Social media facilitates 2-way communication and direct engagement with audiences. Safko and Brake [16] define social media as “activities, practices, and behaviors among communities of people who gather online to share information, knowledge, and opinions using conversational media.” Today, there are over 3.8 billion active social media users worldwide across many different platforms [17]. In the field of health education and promotion, the use of social media has established its effectiveness by providing access to information, delivering health campaigns, and offering social support [18]. Many government agencies and public health organizations (eg, the WHO, the Centers for Disease Control and Prevention [CDC], and other local health departments) have adopted social media to enhance their communication with the public [19].

Social media can serve as a useful tool to relay outbreak-related updates and critical information effectively to the public. Existing research suggests that people often turn to social media for information during infectious disease outbreaks, which can influence their decision-making and subsequent behaviors [19]. The WHO calls for more proactive use of social media to disseminate health messages to journalists, physicians, and the general public, particularly to counteract misinformation regarding infectious diseases [20].

Several studies have investigated the use of social media platforms such as Twitter and Facebook during infectious disease outbreaks. For example, Chen et al [21] studied the temporal variability in the CDC’s response during different stages of the Zika epidemic and public engagement on Twitter. They reported that the CDC was more active in the early warning stages of the Zika epidemic and successfully gained public attention, particularly in the first quarter of 2016. However, when the number of Zika cases increased sharply in the second and third quarters of 2016, the CDC’s efforts on Twitter decreased substantially.

Lwin et al [22] examined the strategic use of Facebook in communicating the Zika epidemic by three main Singapore health authorities: the National Environment Agency, the Health Promotion Board, and the MOH. The researchers found that Facebook was used strategically for Zika-related communication. They also found that preparedness messages (eg, posts mentioning responders and providing recommendations to reduce harm) may have been the most effective, as evidenced by greater levels of public engagement.

Guidry et al [23] examined Ebola-related posts on Instagram and Twitter from three key health organizations: the CDC, the WHO, and Médecins Sans Frontières (ie, Doctors Without Borders). They found Instagram to be a particularly useful platform for communicating with the public during crises. It was further suggested that social media messaging is more effective when it is utilized by health organizations with which the public is already familiar, and when it is based on strategic use of risk communication principles.

A recent study by Raamkumar [24] examined the use of Facebook for COVID-19–related outreach by public health authorities in Singapore, the United States, and England, and the corresponding public response to these efforts. They reported that the Singapore MOH was the most active in terms of posting frequency, while the CDC elicited the most responses. Furthermore, they reported that posts on preventive and safety measures and situation updates were the most frequently employed by public health authorities in these 3 countries.

### Theoretical Framework

Crisis events such as the COVID-19 pandemic requires unique health communication and education strategies in which public health authorities must meet the public needs for information [14]. Theories suggests that the public has various information needs at different stages of a crisis [25]. The Crisis and Emergency Risk Communication (CERC) model serves as a useful tool to guide authorities’ communication strategies during different stages of a crisis. It specifies a broad set of communication activities that vary throughout the life cycle of the crisis. The CERC model was originally developed by the CDC after the 2001 anthrax attacks and the events of September 11, 2001, in the United States [26,27]. It is an integrated model that draws elements from risk communication theories (persuading individuals to take action to limit risks), crisis communication theories (responding to the public’s immediate need for information), and theories of health communication [27].

The CERC model describes five general stages of a crisis: precrisis, initial event, maintenance, resolution, and evaluation. For each stage, a set of recommended communication activities is also described. According to the model, specific and distinct communication activities should be carried out in each stage.

The first stage of the CERC model is the precrisis period. In this stage, the crisis has yet to occur. Communication messages at this stage should focus on risk information, warnings, and preparation.

The second stage of the model is the initial event, when the crisis actually occurs. This stage is initiated by a clear trigger event that signals the beginning of a crisis. Communication messages in this stage should focus on reducing public uncertainty by providing timely updates regarding the crisis, messages of self-efficacy, and reassurance from authority-initiated measures.

The third stage, or maintenance stage, begins when “most or all of the direct harm is contained, and the intensity of the crisis begins to subside” [28]. It further echoes many of the communication activities from earlier stages, including uncertainty reduction, reassurance, and self-efficacy messages.

The fourth stage is the resolution stage, in which the crisis continues to wind down and new understandings of risk emerge. Communication messages at this stage involve updates about ongoing resolutions, discussions about causes, and new understandings of risk.

The final stage is the evaluation stage. This stage occurs when the crisis itself is over. Communication during this stage should focus on assessing the adequacy and efficacy of the response and reaching a consensus on the lessons learned from the crisis [26,28].

### Objective

With over 15 million Twitter users in Saudi Arabia [29], the microblogging and social media platform Twitter presents an opportunity to examine the Saudi MOH’s use of social media in crisis communication during pandemics. According to a recent national survey, approximately 78% of respondents reported the MOH as their main source of information about COVID-19 [30]. The objective of this study is to investigate the use of Twitter by the MOH and the associated public engagement during different stages of the COVID-19 pandemic in Saudi Arabia.

## Methods

### Data Collection

All tweets from the Saudi MOH (@SaudiMOH) posted between January 1 and August 31, 2020, were collected and included in the study. The tweets were collected on September 30, 2020, via the GET statuses/user_timeline endpoint of Twitter’s application programming interface [31] by using the python library Tweepy [32]. For each tweet, the following data were collected: tweet ID, tweet text (body), number of likes, number of retweets, and date posted. Only original tweets, rather than retweets, were considered. Tweets written in languages other than Arabic or English were removed to avoid misinterpretation. Tweets unrelated to COVID-19 were manually excluded by scanning the content of the tweets. Daily confirmed case counts of COVID-19 in Saudi Arabia were obtained from Our World in Data [33].

### Crisis Stages

The various stages of the CERC model have not been clearly defined or operationalized within the context of infectious disease outbreaks. The CERC model assumes that “crises will develop in largely predictable and systematic ways” [26]. However, infectious disease outbreaks such as the current COVID-19 pandemic may last for months or even years, without clear boundaries (compared to other crises, such as extreme weather events) [34,35]. In this study, outbreak stages were determined on the basis of the CERC model and specific events of the COVID-19 outbreak in Saudi Arabia.

The precrisis stage was determined to span from January 1 to March 1, 2020, when the outbreak began in China and some European countries, but when no cases had yet been identified in Saudi Arabia.

The initial event stage was determined to last from March 2 to June 20, 2020. The first confirmed case of COVID-19 in Saudi Arabia was reported on March 2. During this period, there were 157,600 confirmed cases of COVID-19 in the country.

The maintenance stage was determined to span from June 21 to August 31, 2020. On June 21, 2020, Saudi Arabian authorities lifted the nationwide curfew and allowed the resumption of all activities, except in Mecca.

### Content Analysis and Coding Categories

This study used a content analysis approach to investigate the Saudi MOH’s communication on Twitter regarding the COVID-19 pandemic. Berg [36] explained that content analysis is a “careful, detailed, systematic examination and interpretation of a particular body of material in an effort to identify patterns, themes, biases, and meanings.” COVID-19–related tweets were coded using a codebook adapted from one that was developed on the basis of the CERC framework [22]. The codebook consisted of the following categories: (1) risk messages, including tweets containing information about the disease, its transmission mechanisms, and its symptoms; (2) warnings, including tweets highlighting risk factors and dangers associated with COVID-19; (3) preparations, including tweets mentioning responders and providing response recommendations and advice; (4) uncertainty reduction, including tweets containing information about case reports and providing the public with reliable sources of information; (5) efficacy, including tweets containing information about personal preventive measures and highlighting the common responsibility for disease prevention; and (6) reassurance, including tweets that calmed the public by providing information about government interventions and expressing gratitude and regards to the health staff and the public. Through initial scanning of the tweets, digital responses were found to be frequently mentioned in the MOH tweets. Given the importance of digital health during the COVID-19 pandemic, an additional category called “digital health responses” was introduced. Messages of this category pertained to tweets promoting digital health services, ranging from digital screening to surveillance, contact tracing, and follow-up apps. The codebook is presented in Multimedia Appendix 1.

Each tweet was primarily categorized on the basis of the content within its 280 characters. If the content was not clear, linked visuals such as photographs, videos, and other media were analyzed. It was beyond the scope of this study to analyze the content of hyperlinks. The content analysis of the MOH’s tweets was conducted using Excel (version 16.43, Microsoft Inc), which was later imported into SPSS (version 27, IBM Corp) for statistical analysis.

### Intercoder Reliability

Intercoder reliability was established by 2 independent coders (FH and SD). Each author independently coded a randomly selected subsample of 122 (10%) tweets. This meets the Neuendorf recommendation of coding 10%-20% of the total sample for reliability [37]. Reliability was assessed using the ReCal statistical program with the Cohen κ statistic [38]. The κ values for all categories were greater than 0.8, which indicated “almost perfect” agreement, except for the coding category of “Responders,” which had a κ value of 0.545, indicating “moderate agreement” [39]. Coding discrepancies for the “Responders” category were resolved through discussion. Once intercoder reliability was established, the first coder coded the remaining tweets.

### Statistical Analysis

Statistical analysis was performed using SPSS (version 27) [40]. Counts and percentages were used to summarize categorical variables. The median was used to summarize the distribution of continuous variables owing to the skewed nature of those included. The chi-square test of independence was used to assess the associations between outbreak stages and message types. Significant chi-square outcomes were further subjected to multiple post hoc *Z*-tests to compare each pair of outbreak stages. *P* values were adjusted for the false discovery rate by using the Benjamini-Hochberg adjustment.

The nonparametric Kruskal–Wallis test was used to compare the distribution of likes and retweets (engagement indicators) between outbreak stages. The Mann–Whitney *U* test was used to test differences in engagement between tweets including and those not including different message types.

A negative binomial regression analysis was used to examine associations among tweet content, media type, crisis stage, and public engagement. Negative binomial regression was used as engagement variables demonstrated positive skew and overdispersion. The incidence rate ratio (IRR) was calculated as the exponent of the regression coefficients. All statistical tests were performed at a significance level of .05.

### Ethics Approval

Ethics approval was not required for this study as the study did not involve any human subjects. All data analyzed in this study were publicly available and collected from a governmental public Twitter account.

### Data Availability

The data that support our findings are available on request from the corresponding author.

## Results

### Results Overview

The MOH posted a total of 1393 original tweets (an average of 5.85 tweets per day) during the study period. Overall, 1293 (92.8%) tweets were related to COVID-19, of which 1217 were included in the analysis. The other tweets (n=76) were removed because they were in languages other than Arabic or English. The results are presented in three sections: the MOH response to COVID-19 on Twitter across stages, message types across stages, and public engagement with tweets from the MOH.

### MOH Response to COVID-19 on Twitter Across Stages of the COVID-19 Outbreak

**Figure 1:**
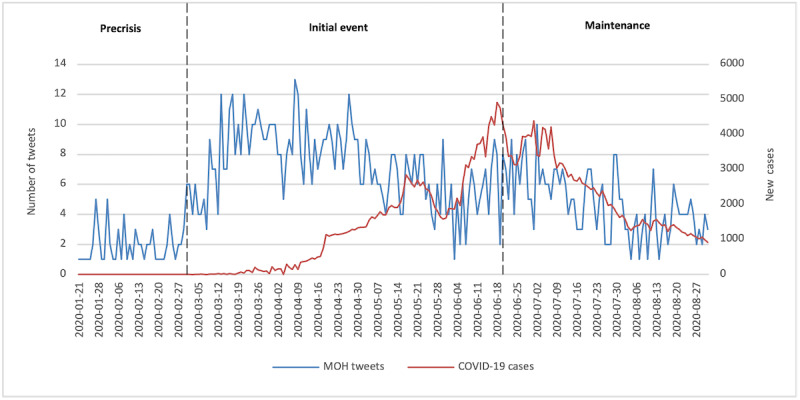
The Saudi Ministry of Health's Twitter communication in relation to confirmed COVID-19 cases (January 1 to August 31, 2020). MOH: Ministry of Health.

Confirmed and reported COVID-19 cases were plotted in relation to COVID-19–related tweets posted by the MOH (Figure 1). The first COVID-19–related tweet was posted on January 21, 2020. Overall, 79 (6.5%) tweets were posted during the precrisis stage, when the outbreak began in China and some European countries, but when no cases had yet been identified in Saudi Arabia (2.03 tweets per day on average). On March 2, 2020, the MOH confirmed the first COVID-19 case in Saudi Arabia, which signaled the start of the crisis. COVID-19–related tweets were consistently posted as the number of cases increased, with an average of 7.23 daily tweets. As of June 20, 2020, the MOH had posted 802 tweets (66% of the total), which is a >3-fold increase in its average daily tweets compared to the precrisis stage. On June 21, 2020, the country lifted curfew restrictions and resumed all economic and commercial activities [41]. From that date until the end of August 2020, the MOH continued to provide ongoing information regarding COVID-19, with an average of 4.67 daily tweets (n=336, 27.6%).

### Message Types Across Stages of the COVID-19 Outbreak

Message types across stages of the outbreak are summarized in Table 1. Of the 1217 tweets, nearly half (49.47%) contained uncertainty reduction information, 28.35% contained efficacy information, and one-fifth (21.53%) of all tweets contained reassuring information.

Tweets about warning messages accounted for a low proportion of all tweets in the precrisis stage (2.5%), which significantly increased during the initial stage (*χ^2^_2_*=6.2; *P=*.046) (Table 1 and Multimedia Appendix 2). Tweets about preparation messages accounted for a low proportion of tweets in the precrisis stage (11.4%) and the initial stage (11.2%), and the proportion increased significantly in the maintenance stage (22.3%; *χ^2^_2_*=24.4; *P*<.001). Conversely, the percentage of reassurance tweets peaked in the precrisis stage (67.1%) and significantly decreased in later stages (*χ^2^_2_*=103.9; *P*<.001). The frequency of efficacy tweets was higher in the initial stage (34.4%) than in the precrisis (15.2%) and maintenance (16.9%) stages (*χ^2^_2_*=42.7; *P*<.001). Lastly, tweets promoting digital health services increased significantly in frequency, from 8.6% in the initial stage to 16.7% in the maintenance stage (*χ^2^_2_*=26.4; *P*<.001). 

**Table 1:** Categories of message types across outbreak stages in Saudi Arabia (January 1 to August 31, 2020).

Message type^a^		Precrisis stage (n=79), n (%)	Initial event stage (n=802), n (%)	Maintenance stage (n=336), n (%)	Overall (n=1217), n (%)	*P* value	*χ^2^* (*df*=2)
**Risk messages**		5 (6.3)	62 (7.7)	21 (6.3)	88 (7.2)	.65	0.876
	Disease information	4 (5.1)	24 (3.0)	8 (2.4)	36 (3.0)	.45	1.613
	Symptoms	3 (4.8)	44 (5.5)	15 (4.5)	62 (5.1)	.67	0.805
**Warnings**		2 (2.5)	91 (11.3)	33 (9.8)	126 (10.4)	.046	6.162
	Risk factor	2 (2.5)	40 (5.0)	29 (8.6)	71 (5.8)	.03	7.399
	Danger	0 (0.0)	53 (6.6)	4 (1.2)	57 (4.7)	<.001	19.722
**Preparations**		9 (11.4)	90 (11.2)	75 (22.3)	174 (14.3)	<.001	24.390
	Responders	8 (10.1)	27 (3.4)	11 (3.3)	46 (3.8)	.009	9.363
	Recommendations	1 (1.3)	64 (8.0)	64 (19.0)	129 (10.6)	<.001	38.376
**Uncertainty reduction**		41 (51.9)	382 (47.6)	179 (53.3)	602 (49.5)	.20	3.216
	Case report	30 (38.0)	157 (19.6)	72 (21.4)	259 (21.3)	.001	14.538
	Information resources	12 (15.2)	225 (28.1)	107 (31.8)	344 (28.3)	.01	8.802
**Efficacy**		12 (15.2)	276 (34.4)	57 (17.0)	345 (28.3)	<.001	42.699
	Personal prevention	12 (15.2)	226 (28.2)	51 (15.2)	289 (23.7)	<.001	25.520
	Common responsibility	0 (0.0)	86 (10.7)	9 (2.7)	95 (7.8)	<.001	28.447
**Reassurance**		53 (67.1)	145 (18.1)	64 (19.0)	262 (21.5)	<.001	103.938
	Calming	51 (64.6)	91 (11.3)	41 (12.2)	183 (15)	<.001	162.297
	Thanks and regards	0 (0.0)	41 (5.1)	23 (6.8)	64 (5.3)	.047	6.117
	Government interventions	4 (5.1)	57 (7.1)	26 (7.7)	87 (7.1)	.71	0.696
Digital health responses		0 (0.0)	69 (8.6)	56 (16.7)	125 (10.3)	<.001	26.375

^a^A tweet can have more than 1 category.

### Public Engagement With MOH Tweets

**Figure 2:**
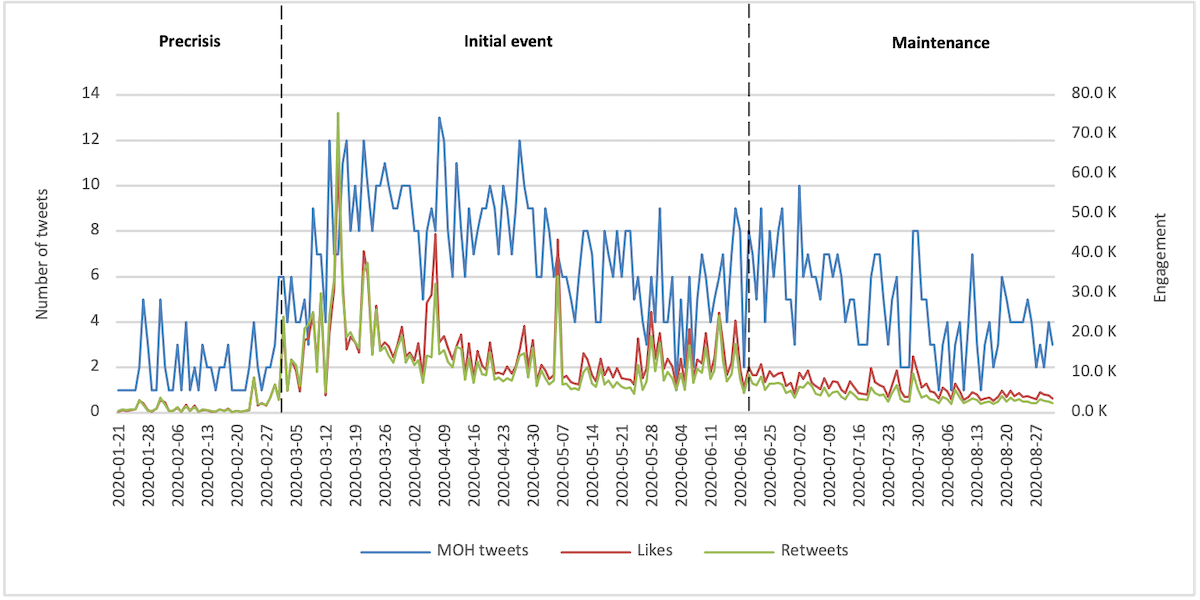
Public engagement in relation to the Saudi Ministry of Health's tweets (January 1 to August 31, 2020). The left y-axis shows the daily number of tweets. The right y-axis shows the number of likes and retweets (in thousands). MOH: Ministry of Health.

Figure 2 demonstrates public engagement (represented by frequencies of likes and retweets) in relation to MOH tweets. Public engagement was not accurately aligned with the development of the COVID-19 outbreak in the country; rather, the public was most engaged when the initial cases appeared in March 2020.

Since engagement variables were not normally distributed, median values (rather than mean values) were used for statistical comparisons. MOH tweets were associated with median values of 819 likes and 603 retweets. A Kruskal–Wallis *H* test revealed significant differences in the frequencies of likes (*χ^2^_2_*=70.344; *P*<.001) and retweets (*χ^2^_2_*=59.764; *P*<.001) among different outbreak stages. The Mann–Whitney *U* test was used for post hoc comparisons of average ranks. Post hoc pairwise comparisons (Multimedia Appendix 3) revealed that the distribution of retweets was not significantly different between the precrisis and maintenance stages (*Z*=–2.14; *P*=.96).

Median public engagement levels across different stages of the outbreak and message types are summarized in Table 2. Overall, the public engaged most with tweets from the MOH during the initial stage, with median values of 974 likes and 753 retweets. Further analysis using Mann–Whitney *U* tests (Table 3) revealed that both like and retweet frequencies during the initial stage were significantly higher for tweets that provided uncertainty reduction messages (*Z*=–3.133; *P*=.002) and reassurance messages (*Z*=–5.843; *P*<.001) than those that did not. Tweets containing risk messages at this stage received fewer likes (*Z*=–4.219; *P*<.001) and retweets (*Z*=–4.252; *P*<.001) than those that did not. In contrast, tweets containing risk messages during the precrisis stage received more likes (*Z*=–2.034; *P*=.04) and retweets (*Z*=–2.15; *P*=.03) than those that did not. Among tweets in the maintenance stage, the frequencies of both likes (*Z*=–3.708; *P*<.001) and retweets (*Z*=–3.605; *P*<.001) were significantly higher for tweets that contained uncertainty reduction information than for those that did not. Conversely, frequencies of both likes (*Z*=–4.534; *P*<.001) and retweets (*Z*=–4.547; *P*<.001) were significantly lower for tweets that promoted digital health services than for those that did not.

**Table 2:** Median public engagement across outbreak stages and message types (January 1 to August 31, 2020).

Message type^a^	Likes					Retweets			
	All stages	Precrisis stage	Initial event stage	Maintenance stage	All stages		Precrisis stage	Initial event stage	Maintenance stage
All	819.0	437.0	974.0	582.0	603.0		473.0	753.0	351.0
Risk messages	598.5	686.0	475.5	630.0	409.5		766.0	385.5	381.0
Warnings	785.0	1678.0	913.0	513.0	614.5		1801.0	710.0	335.0
Preparations	594.5	343.0	974.0	464.0	397.5		438.0	708.0	273.0
Uncertainty reduction	841.5	389.0	1025.0	678.0	658.5		438.0	770.0	409.0
Efficacy	852.0	560.0	950.5	590.0	683.0		623.5	755.5	422.0
Reassurance	982.0	418.0	1629.0	568.5	740.0		401.0	1198.0	316.0
Digital health responses	650.0	0.0	899.0	452.0	444.0		0.0	620.0	273.5

^a^Values presented are medians.

**Table 3:** Median public engagement for tweets with or without message type at different stages of the COVID-19 outbreak in Saudi Arabia (January 1 to August 31, 2020).

Variable^a^				Median present		Median absent		*U* value		*Z*-value		*P* value
**Precrisis**												
	**Risk messages**											
		Retweets	766.00		432.00		78.000		–2.154		.03	
		Likes	686.00		403.50		84.000		–2.034		.04	
**Initial event**												
	**Risk messages**											
		Retweets	385.50		781.50		15490.000		–4.252		<.001	
		Likes	974.50		1008.00		15547.500		–4.219		<.001	
	**Uncertainty reduction**											
		Retweets	770.00		723.50		70667.500		–2.915		.004	
		Likes	1025.00		964.50		69956.000		–3.133		.002	
	**Reassurance**											
		Retweets	1198.00		659.00		34581.000		–5.169		<.001	
		Likes	1629.00		856.00		32880.500		–5.843		<.001	
**Maintenance**												
	**Uncertainty reduction**											
		Retweets	409.00		317.50		10781.500		–3.681		<.001	
		Likes	678.50		524.50		10680.000		–3.795		<.001	
	**Digital health responses**											
		Retweets	273.50		404.50		4823.000		–4.547		<.001	
		Likes	452.00		643.50		4831.000		–4.534		<.001	

^a^Only significant variables are reported.

Negative binomial regression outcomes are summarized in Table 4 (complete models are provided in Multimedia Appendix 4). The model was significantly better than the null model, which indicated that the variables (as a set) predicted the number of likes (n=1217; *χ^2^_12_*=458.627; *P*<.001). The negative binomial regression model for retweets (Table 4) was also significantly better than the null model (n=1217; *χ^2^_12_*=575.495; *P*<.001). The Wald test revealed that all of predictor variables were significant (*P<*.05), except for risk messages and preparations.

Our results indicate that the use of hashtags was significantly associated with higher levels of engagement (likes: IRR=2.470; *P*<.001; retweets: IRR=2.813; *P*<.001), whereas the use of hyperlinks was significantly associated with lower levels of engagement (likes: IRR=0.839; *P*=.045; retweets: IRR=0.727; *P*<.001). Compared to text-only content, the use of photographs and videos was associated with significantly lower numbers of likes and retweets (photographs: likes, IRR=0.530; *P*<.001; retweets, IRR=0.476; *P*<.001; videos: likes, IRR=0.698; *P*=.006; retweets, IRR=0.576; *P*<.006).

With respect to the impact of content type on public engagement, tweets with content related to warnings (likes: IRR=1.334; *P*=.005; retweets: IRR=1.544; *P*<.001), uncertainty reduction (likes: IRR=2.210; *P*<.001; retweets: IRR=2.197; *P*<.001), and reassurance (likes: IRR=1.551; *P*<.001; retweets: IRR=1.517; *P*<.001) were significantly associated with higher levels of engagement.

Regarding crisis stages, tweets posted during the initial and maintenance stages were significantly associated with higher levels of engagement than those posted during the precrisis stage (initial stage: likes, IRR=2.931; *P*<.001; retweets, IRR=2.471; *P*<.001; maintenance stage: likes, IRR=2.355; *P*<.001; retweets, IRR=1.623; *P*<.001).

**Table 4:** Associations of tweet content, media type, and crisis stage with public engagement (January 1 to August 31, 2020).

Variables		Likes				Retweets		
		Incidence rate ratio	95% CI	*P* value	Incidence rate ratio		95% CI	*P* value
Intercept		320.737	227.465-452.255	<.001	325.265		231.247-457.509	<.001
Hashtags		2.470	2.172-2.810	<.001	2.813		2.469-3.206	<.001
Hyperlinks		0.839	0.707-0.996	.045	0.727		0.611-0.865	<.001
**Media type**								
	Text only	Reference	N/A^a^	N/A	Reference		N/A	N/A
	Photographs	0.530	0.428-0.656	<.001	0.476		0.384-0.591	<.001
	Videos	0.698	0.540-0.901	.006	0.576		0.445-0.745	<.001
**Message type**								
	Risk messages	1.177	0.934-1.482	.167	1.088		0.863-1.371	.48
	Warnings	1.334	1.089-1.634	.005	1.544		1.261-1.891	<.001
	Preparations	1.007	0.836-1.212	.944	1.008		0.838-1.213	.93
	Uncertainty reduction	2.210	1.882-2.595	<.001	2.197		1.870-2.583	<.001
	Efficacy	1.096	0.926-1.297	.288	1.200		1.014-1.420	.03
	Reassurance	1.551	1.320-1.821	<.001	1.517		1.296-1.776	<.001
**Crisis stage**								
	Precrisis	Reference	N/A	N/A	Reference		N/A	N/A
	Initial event	2.931	2.309-3.721	<.001	2.471		1.945-3.141	<.001
	Maintenance	2.355	1.825-3.039	<.001	1.623		1.256-2.096	.001

^a^N/A: not applicable.

## Discussion

### Principal Findings

This study shows that Twitter was used as a real-time communication channel to share, communicate, and disseminate information during the COVID-19 pandemic. Overall, the MOH’s response to COVID-19 on Twitter was aligned with the developments of the outbreak in Saudi Arabia. During the precrisis and maintenance stages, the MOH’s use of Twitter was partially consistent with the CERC model. While in the initial stage, the MOH’s communication was in line with the CERC model. The results of public engagement showed that the levels of engagement were different as the pandemic evolved. The tweets in the initial stage elicited the most engagement by far.

The number of tweets pertaining to the COVID-19 pandemic was relatively low (n=79) in the precrisis stage. However, when COVID-19 emerged in Saudi Arabia on March 2, 2020, and began to spread throughout the country, the MOH increased its Twitter activity. The MOH posted most of its COVID-19–related tweets (n=802, 65.89%) during this initial stage. This likely corresponded with the public’s need for information, given that individuals increasingly use social media to seek information during crises [34,42]. The MOH regularly posted tweets regarding COVID-19 (n=336) throughout the maintenance stage and until the end of the study period.

The results concerning public engagement showed that the tweets in the initial stage received the most engagement from the public. This result was expected, considering the lockdowns and curfew restrictions imposed during this stage. Recent studies have even reported an increase in the usage of the internet and social media during COVID-19 lockdowns [43].

The MOH’s use of Twitter was partially consistent with the CERC model. During precrisis, the CERC model suggests that communication should focus on risk information, warnings, and preparations. This is because during the precrisis stage, the public tends to seek information regarding the nature of the risk itself. However, our results show that a large proportion of the MOH’s tweets in this stage included reassurance (67.1%) or uncertainty reduction (51.9%), while those including risk messages and warnings represented only 8.8% of all tweets.

Agwa [44], in her study of the Egyptian MOH’s use of Facebook during the COVID-19 pandemic, also observed a lack of risk information during early stages of the pandemic. One potential explanation for this finding is the novelty and scientific uncertainty associated with COVID-19 [45,46].

During the initial stage, the CERC model suggests that communication messages should focus on reducing public uncertainty and providing messages regarding efficacy and reassurance. Consistent with the model, the MOH reduced uncertainty by updating case reports, holding press conferences, and providing information sources that accounted for 47.6% of tweets. The MOH tweets also emphasized efficacy (34.4%) by highlighting common responsibility and personal prevention measures to limit the spread of SARS-CoV-2 while providing reassurance (18.1%). The MOH’s communication during this stage was in line with the CERC model.

As a crisis continues into the maintenance stage, the CERC model requires ongoing communication of uncertainty reduction and reassurance. Additional efficacy messages inform members of the public about the expected course of action at this stage.

In accordance with the model, the MOH’s tweets during this stage provided information sources to reduce uncertainty in addition to efficacy and reassurance information. However, the results showed that a considerable proportion (22.3%) of the MOH’s tweets in this stage included preparation messages (mostly recommendations). This could potentially be part of the MOH’s efforts to prepare the public for the “new normal,” especially as the country lifted its nationwide curfew and began its gradual reopening during this stage. While the CERC model suggests that health communicators should offer reassurance in the maintenance stage, crisis communication experts do not recommend that messages be overly reassuring, as such information may reduce an authority’s credibility [47]. This is particularly the case in unexpected and unpredictable events such as the COVID-19 pandemic. In addition, it should be noted that some infectious disease outbreaks and epidemics commonly become chronic crises that develop into crisis stages for longer periods, making it difficult to make precise predictions [26].

Most importantly, our findings indicate that different types of messages received different levels of engagement as the outbreak evolved. In the precrisis stage, the public showed a high level of interest in warnings and risk messages, as indicated by a high level of engagement. Based on this finding, it may be inferred that the public wanted to understand the full scope of the risk. In the initial stage, members of the public engaged more with certain messages (such as uncertainty reduction and reassurance) than others; indicating their simultaneous need for increased understanding and reduced anxiety regarding the outbreak. During the maintenance stage, the public also showed a high level of interest in information related to uncertainty reduction, which indicates that they may have still felt uncertain during this stage.

Our findings also identified a number of factors associated with greater public engagement during the pandemic. First, our results show that the use of hyperlinks was negatively associated with public engagement. This further supports the inferences by Chung [48] that hyperlinks increase the complexity of a message by requiring an extra action by the audience, thereby reducing engagement. Another potential explanation is that hyperlinks direct people to another webpage, at which point they may forget about the original message. Second, in contrast with previous reports supporting the positive effect of media on public engagement [49-52], we found that the inclusion of multimedia content (eg, photographs and videos) was negatively associated with public engagement. This finding is similar to that of Chen et al [53], who also found that media richness was negatively associated with public engagement with government social media during the COVID-19 pandemic. This discrepancy can be attributed to the differences in the events examined, as most studies supporting the positive effect were conducted in noncrisis situations [53].

The content of the tweets was also significantly associated with public engagement, where tweets related to uncertainty reduction, public reassurance, and warnings received higher levels of engagement. This is consistent with the results of Tang et al [54], who examined the public health agencies’ tweets in Texas, where tweets that provided information about COVID-19 or described the government’s actions in containing the spread of COVID-19 were found to be more likely to be retweeted. This suggests that as the CERC model indicates, people need this type of information during a public health crisis.

### Limitations

There are several limitations and future considerations of this study. First, while this study focused on Twitter, the MOH used other social media platforms (such as Facebook and Instagram) during the COVID-19 pandemic. Twitter is a microblogging platform that allows users to post short messages that contain up to 280 characters. Facebook allows for much longer posts than Twitter, while Instagram is centered on images rather than text. Since social media platforms vary greatly in their characteristics, different communication strategies may be adopted and employed. Future studies should consider focusing on the same topic on Facebook, Instagram, and other popular social media platforms.

Second, given that the pandemic is still ongoing, this study focused on the first 3 stages of the crisis (precrisis, initial, and maintenance stages) and did not examine the resolution or evaluation stage. Future studies should expand the scope of the analysis to provide a more comprehensive description of the MOH’s crisis communication on Twitter.

Only tweets written in Arabic and English were included in the analysis. In addition, this study did not attempt to analyze public replies to the MOH’s tweets. Further studies are needed to examine the content of the public’s replies to understand their responses and opinions regarding tweets from the MOH. A final limitation is the time interval between the posting date of a tweet and the date of data collection. Since older tweets may take a longer time to accumulate engagement, future studies should consider such temporal effects. Future studies should also incorporate more appropriate measures of engagement, beyond simply the numbers of likes and retweets.

### Conclusions

The COVID-19 pandemic is an extreme crisis and has generated significant challenges for governments. Effective communication with the public is of crucial importance. This study provided some insight into the Saudi MOH’s outbreak communication strategy. Our findings identified differences in MOH communication practices during different stages of the COVID-19 outbreak in Saudi Arabia, in terms of both types of message content and levels of public engagement. Uncertainty reduction, efficacy, and reassurance were the most common types of messages in MOH tweets. Our results provide several implications for crisis communication by researchers, governments, health organizations, and practitioners to engage their external public. Governments and health authorities should consider the public’s information needs to promote their engagement; this, in turn, could raise the public’s awareness of a health crisis. Effective communication during disease outbreaks and other public health emergencies has the potential to change outcomes and save lives. 

## Appendix

Multimedia Appendix 1 Codebook.

Multimedia Appendix 2 Categories of message types by outbreak stages, post-hoc pairwise comparisons.

Multimedia Appendix 3 Public engagement across outbreak stages, post-hoc pairwise comparisons.

Multimedia Appendix 4 Negative binomial regression results for associations of tweet content, media type, and the crisis stage with public engagement.

## Abbreviations

CDC: Centers for Disease Control and Prevention
CERC: Crisis and Emergency Risk Communication
IRR: incidence rate ratio
MOH: Ministry of Health
WHO: World Health Organization
