# Supplementary material for: The Saudi Ministry of Health’s Twitter Communication Strategies and Public Engagement During the COVID-19 Pandemic: Content Analysis Study
Source: JMIR Public Health Surveill. 2021 Jul 12;7(7):e27942. doi: 10.2196/27942 (PMC8276783; doi:10.2196/27942)
Supplement: Multimedia Appendix 1 [file publichealth_v7i7e27942_app1.docx]

**Multimedia Appendix 1.** Codebook.

| **Theme category** | **Definition** | **Example** |
| --- | --- | --- |
| ***Risk messages*** |  |  |
| Symptoms | Statements on symptoms associated with COVID-19 | #اعزل_نفسك عن عائلتك ! فهي أول خطوة عند شعورك بأعراض #كورونا : " كحة، ارتفاع في الحرارة، ضيق بالتنفس". وكلم الصحة 937 📞 <https://t.co/2BCOSBMbah> |
| Disease information | General information about the disease | ماهو فيروس كورونا الجديد ؟ <https://t.co/1gsGBZckvW> |
| ***Warnings*** |  |  |
| Risk factors | Statements on risk factors or risk groups associated with COVID-19 | من هم الفئات الأكثر خطورة للإصابة بفيروس #كورونا؟ هُنا الإجابة ⬇ <https://t.co/3G5Ty2pEQv> |
| Danger | Statement that highlights the risk of COVID-19 | عدوى #كورونا سريعة الانتشار لذلك #خلك_بالبيت ولا تتهاون. #كلنا_مسؤول <https://t.co/T1onbSiehL> |
| ***Preparations*** |  |  |
| Responders | Organizations or persons who will be responsible for the emergency management/ coordination among responder groups | عقدت اللجنة المعنية بمتابعة الوضع الصحي لفيروس [#كورونا_الجديد](https://twitter.com/hashtag/%D9%83%D9%88%D8%B1%D9%88%D9%86%D8%A7_%D8%A7%D9%84%D8%AC%D8%AF%D9%8A%D8%AF?src=hashtag_click) مؤتمراً صحفياً اليوم الأحد استعرضت من خلاله الإجراءات والإستعدادات المتخذة لمواجهة الفيروس. |
| Recommendations | Requests and advises on taking actions to prevent COVID-19 | من أجل سلامتكم ننصح بتأجيل المواعيد والإجراءات الطبية غير الملحة. [#الوقاية_من_كورونا](https://twitter.com/hashtag/%D8%A7%D9%84%D9%88%D9%82%D8%A7%D9%8A%D8%A9_%D9%85%D9%86_%D9%83%D9%88%D8%B1%D9%88%D9%86%D8%A7?src=hashtag_click) |
| ***Uncertainty reduction*** |  |  |
| Case reports | Reports and updates of cases | ##الصحة تعلن عن تسجيل (92) حالة إصابة جديدة بفيروس #كورونا الجديد (كوفيد19)، وتسجيل حالتي تعافي ليصبح مجموع الحالات المتعافية (35) حالة ولله الحمد. <https://t.co/p1VS1PBBUI> |
| Information resources | sources that allow people to learn more about COVID-19 | كل ماتريد معرفته من معلومات عن فيروس #كورونا الجديد في هذا الملف ⬇️ <https://t.co/QAEDXb0G4G> |
| ***Efficacy*** |  |  |
| Personal prevention measures | Specific prevenetion actions one can take to prevent COVID-19 | تعرف على طرق الوقاية من فيروس #كورونا الجديد [https://t.co/ojp0Cpdrni](https://t.co/ojp0CPdrni) |
| Common responsibility | Exprssion of common responsibility for the public and other stakeholders | نلتزم جميعًا بالبقاء في المنزل وترك مسافة آمنة. #الوقاية_من_كورونا #كلنا_مسؤول <https://t.co/RacIyuGjir> |
| ***Reassurance*** |  |  |
| Calming | Statements that remove uncertainty or fears of the COVID-19 threat | بفضل الله لم تسجل حتى الآن أي حالة إصابة بفيروس #كورونا الجديد داخل المملكة، ويتم تطبيق الإجراءات الإحترازية والوقائية في المنافذ لمنع وفادة الفيروس إلى المملكة. <https://t.co/0mcwbYqFFx> |
| Government interventions | Government intervention responses to COVID-19 | المسح النشط للأحياء في مكة المكرمة والمدينة المنورة. #كلنا_مسؤول #الوقاية_من_كورونا <https://t.co/WOmw471x79> |
| Thanking and regards | Exprssion of thanks, approval and regards | آلاف الورود، محفوفة بالشكر والتقدير، قدمناها للملتزمين بالإجراءات الوقائية، وزعت بالمحبة في أكثر من منطقة في وطننا الغالي. <https://t.co/JMRvVY49Ca> |
| ***Digital health responses*** | Mention of digital health applications such as Mawid, Sehhaty, Tabaud, Tetamman, and Tawakkalna | [القضاء على جائحة كورونا يحتاج تضافر جهود الجميع، تحميلك #تطبيق_توكلنا يساعد العاملين في المجال الصحي على القيام بواجبهم، كما يساعد الجهات الأخرى على القيام بدورها في مكافحة الجائحة.](https://t.co/eu9ka9iO1l)  [رابط التحميل:](https://t.co/eu9ka9iO1l)  <https://t.co/eu9ka9iO1l> |
