# Supplementary material for: The Saudi Ministry of Health’s Twitter Communication Strategies and Public Engagement During the COVID-19 Pandemic: Content Analysis Study
Source: JMIR Public Health Surveill. 2021 Jul 12;7(7):e27942. doi: 10.2196/27942 (PMC8276783; doi:10.2196/27942)
Supplement: Multimedia Appendix 2 [file publichealth_v7i7e27942_app2.docx]

**Multimedia Appendix 2.** Categories of message types by outbreak stages, post-hoc pairwise comparisons.

| Message type | Precrisis stage | Initial event stage | Maintenance stage |
| --- | --- | --- | --- |
|  | (A) | (B) | (C) |
| **Risk messages** |  |  |  |
| Disease information |  |  |  |
| Symptoms |  |  |  |
| **Warnings** |  | **A( .045)** |  |
| Risk factor |  |  |  |
| Danger |  | C( .000) |  |
| **Preparations** |  |  | **A( .044)**  **B( .000)** |
| Responders | B( .010)  C( .013) |  |  |
| Recommendations |  | A( .029) | A( .000)  B( .000) |
| **Uncertainty Reduction** |  |  |  |
| Case report | B( .000)  C( .003) |  |  |
| Information resources |  | A( .021) | A( .010) |
| **Efficacy** |  | **A( .001)**  **C( .000)** |  |
| Personal prevention |  | A( .020)  C( .000) |  |
| Common responsibility |  | C( .000) |  |
| **Reassurance** | **B( .000)**  **C( .000)** |  |  |
| Calming | B( .000)  C( .000) |  |  |
| Thanks & regards |  |  |  |
| Government interventions |  |  |  |
| **Digital health responses** |  |  | **B( .000)** |

Results are based on two-sided tests. For each significant pair, the key of the category with the smaller column proportion appears in the category with the larger column proportion. Significance level for upper case letters (A, B, C): .05. Tests are adjusted for all pairwise comparisons within a row of each innermost subtable using the Benjamini-Hochberg correction.
