# Supplementary material for: The Saudi Ministry of Health’s Twitter Communication Strategies and Public Engagement During the COVID-19 Pandemic: Content Analysis Study
Source: JMIR Public Health Surveill. 2021 Jul 12;7(7):e27942. doi: 10.2196/27942 (PMC8276783; doi:10.2196/27942)
Supplement: Multimedia Appendix 3 [file publichealth_v7i7e27942_app3.docx]

**Multimedia Appendix 3.** Public engagement across outbreak stages, post-hoc pairwise comparisons.

|  | Likes | | | | | Retweets | | | |
| --- | --- | --- | --- | --- | --- | --- | --- | --- | --- |
|  | Test statistic | Std. error | Std. test statistic | Adj. sig. | Test statistic | | Std. error | Std. test statistic | Adj. sig. |
|  |  |  |  |  |  | |  |  |  |
| **Precrisis–Maintenance** | -171.452 | 43.946 | -3.901 | < 0.001 | 2.139 | | 43.946 | 0.049 | 1.000 |
| **Precrisis–Initial** | -296.604 | 41.444 | -7.157 | < 0.001 | -162.563 | | 41.444 | -3.922 | < 0.001 |
| **Maintenance–Initial** | 125.152 | 22.840 | 5.480 | < 0.001 | 164.702 | | 22.840 | 7.211 | < 0.001 |
