# Supplementary material for: The Saudi Ministry of Health’s Twitter Communication Strategies and Public Engagement During the COVID-19 Pandemic: Content Analysis Study
Source: JMIR Public Health Surveill. 2021 Jul 12;7(7):e27942. doi: 10.2196/27942 (PMC8276783; doi:10.2196/27942)
Supplement: Multimedia Appendix 4 [file publichealth_v7i7e27942_app4.docx]

**Multimedia Appendix 4**. Negative binomial regression results for associations of tweet content, media type, and the crisis stage with public engagement.

Table1. Results of negative binomial regression for the number of likes

| Variables | B | Exp(B) | SE | *P* value |
| --- | --- | --- | --- | --- |
| (Intercept) | 5.771 | 320.737 | 0.1753 | <.001 |
| Hashtags | 0.904 | 2.470 | 0.0656 | <.001 |
| Hyperlinks | -0.175 | 0.839 | 0.0876 | .045 |
| **Media type** |  |  |  |  |
| Text only | Reference |  |  |  |
| Photos | -0.635 | 0.530 | 0.1089 | <.001 |
| Videos | -0.360 | 0.698 | 0.1305 | .006 |
| **Message type** |  |  |  |  |
| Risk messages | 0.163 | 1.177 | 0.1178 | .167 |
| Warnings | 0.288 | 1.334 | 0.1034 | .005 |
| Preparations | 0.007 | 1.007 | 0.0946 | .944 |
| Uncertainty reduction | 0.793 | 2.210 | 0.0819 | <.001 |
| Efficacy | 0.091 | 1.096 | 0.0860 | .288 |
| Reassurance | 0.439 | 1.551 | 0.0821 | <.001 |
| **Crisis stage** |  |  |  |  |
| Precrisis | Reference |  |  |  |
| Initial event | 1.075 | 2.931 | 0.1217 | <.001 |
| Maintenance | 0.856 | 2.355 | 0.1301 | <.001 |

Table2. Results of negative binomial regression for the number of retweets

| Variables | B | Exp(B) | SE | *P* value |
| --- | --- | --- | --- | --- |
| (Intercept) | 5.785 | 325.265 | 0.1741 | <.001 |
| Hashtags | 1.03 | 2.813 | 0.0666 | <.001 |
| Hyperlinks | -0.319 | 0.727 | 0.0888 | <.001 |
| **Media type** |  |  |  |  |
| Text only | Reference |  |  |  |
| Photos | -0.742 | 0.476 | 0.1099 | <.001 |
| Videos | -0.552 | 0.576 | 0.1316 | <.001 |
| **Message type** |  | 1.544 |  | <.001 |
| Risk messages | 0.084 | 1.088 | 0.1181 | .476 |
| Warnings | 0.434 | 1.544 | 0.1034 | <.001 |
| Preparations | 0.008 | 1.008 | 0.0944 | .931 |
| Uncertainty reduction | 0.787 | 2.197 | 0.0824 | <.001 |
| Efficacy | 0.182 | 1.200 | 0.0860 | .034 |
| Reassurance | 0.417 | 1.517 | 0.0803 | <.001 |
| **Crisis stage** |  |  |  |  |
| Precrisis | Reference |  |  |  |
| Initial event | 0.905 | 2.471 | 0.1223 | <.001 |
| Maintenance | 0.484 | 1.623 | 0.1305 | <.001 |
